# Genome-wide DNA methylome reveals the dysfunction of intronic microRNAs in major psychosis

**DOI:** 10.1186/s12920-015-0139-4

**Published:** 2015-10-14

**Authors:** Hongying Zhao, Jinyuan Xu, Lin Pang, Yunpeng Zhang, Huihui Fan, Ling Liu, Tingting Liu, Fulong Yu, Guanxiong Zhang, Yujia Lan, Jing Bai, Xia Li, Yun Xiao

**Affiliations:** College of Bioinformatics Science and Technology, Harbin Medical University, Harbin, Heilongjiang China; Key Laboratory of Cardiovascular Medicine Research, Harbin Medical University, Ministry of Education, Harbin, Heilongjiang China

**Keywords:** Major psychosis, MeDIP-seq, RNA-seq, DNA methylation, miRNA

## Abstract

**Background:**

DNA methylation is thought to be extensively involved in the pathogenesis of many diseases, including major psychosis. However, most studies focus on DNA methylation alteration at promoters of protein-coding genes, despite the poor correlation between DNA methylation and gene expression.

**Methods:**

We analyzed differentially methylated regions and differentially expressed genes in patients with schizophrenia and bipolar disorder and normal subjects. Gene expression and DNA methylation were analyzed with RNA-seq and MeDIP-seq of post-mortem brain tissue (brain region BA9) cohort in five schizophrenia, seven bipolar disorder cases and six controls, respectively.

**Results:**

Here, we performed a large-scale integrative analysis using MeDIP-seq, coupled with RNA-seq, on brain samples from major psychotic and normal subjects and observed obvious discrepancy between DNA methylation and gene expression. We found that differentially methylated regions (DMRs) were distributed across different types of genomic elements, especially introns. These intronic DMRs were significantly enriched for diverse regulatory elements, such as enhancers and binding sites of certain transcriptional factors (e.g., Pol3). Notably, we found that parts of intronic DMRs overlapped with some intragenic miRNAs, such as hsa-mir-7-3. These intronic DMR-related miRNAs were found to target many differentially expressed genes. Moreover, functional analysis demonstrated that differential target genes of intronic DMR-related miRNAs were sufficient to capture many important biological processes in major psychosis, such as neurogenesis, suggesting that miRNAs may function as important linkers mediating the relationships between DNA methylation alteration and gene expression changes.

**Conclusions:**

Collectively, our study indicated that DNA methylation alteration could induce expression changes indirectly by affecting miRNAs and the exploration of DMR-related miRNAs and their targets enhanced understanding of the molecular mechanisms underlying major psychosis.

**Electronic supplementary material:**

The online version of this article (doi:10.1186/s12920-015-0139-4) contains supplementary material, which is available to authorized users.

## Background

Schizophrenia (SZ) and bipolar disorder (BD), together termed “major psychosis”, could be etiologically correlated psychiatric conditions [1], characterized by long-lasting behavioral abnormalities. Previous studies of major psychosis mainly focused on the genetic susceptibility [2]. However, several epidemiological and clinical peculiarities, such as noncomplete concordance between monozygotic twins and a fluctuating disease course, are still difficult to explain with a small number of risk genes/locis and extremely rare structural variants. Accumulating evidence shows that alterations of DNA methylation, which accounts for most of the silencing events in the genome (such as, X-chromosome inactivation and genomic imprinting [3]), can contribute to the pathogenesis of human diseases. Such aberrant DNA methylation has been recently implicated in the pathogenesis of major psychosis with a growing body of evidence [4–6]. Preliminary investigations of DNA methylation mainly focused on disease-related genes, such as *RELN* [7], *SOX10* [8], *MB-COMT* [9] and *GAD1* [10], which exhibited methylation alterations between major psychosis and normal controls. Subsequently, Mill et al. comprehensively scanned DNA methylation level using microarray technology to systematically identify DNA-methylation changes in the frontal cortex of major psychosis patients [11]. Recently, based on high-throughput sequencing technology, genome-wide DNA methylation analysis combined with transcription changes provides a more effective approach to delineate the molecular mechanisms underlying major psychosis [12].

Nevertheless, the association between DNA methylation and gene expression is still ambiguous and controversial. A number of studies demonstrated that high DNA methylation levels at CpG-rich promoters are incompatible with gene activation [13]. The relationship between DNA methylation and transcription is more nuanced than ever expected, depending on different genomic contexts [14]. Moreover, comparative analysis in cancers [15] uncovered that differences in DNA methylation only resulted in expression changes of a very low percentage of genes. Such inconsistency between DNA methylation and gene expression may imply the existence of other factors bridging them.

At present, most DNA methylation studies focus on identification of aberrant methylation-induced protein-coding genes with differential expression [16]. Indeed, protein-coding genes only constitute a small proportion of the genome, and various regulators were required for precise control of their expressions, such as microRNAs (miRNAs) [17–19] and transcription factors (TFs) [20, 21]. A mountain of evidence has suggested that multiple miRNAs were also involved in the pathogenesis of major psychosis [22]. For instance, Kim et al. [23] investigated the expression of 667 miRNAs in the prefrontal cortex of individuals with schizophrenia and bipolar disorder and then identified 22 differentially expressed miRNAs. These differentially expressed miRNAs were found to target brain specific genes enriched for SZ and BD disease development. Therefore, integration of DNA methylome and transcriptome, and consideration of DNA methylation-induced global effects are necessary to explore how aberrant DNA methylation contributes to the mechanisms underlying major psychosis.

In this study, the DNA methylome and transcriptome maps of brain samples generated from 5 SZ, 7 BD and 6 normal subjects were determined using methylated DNA immunoprecipitation and sequencing (MeDIP-seq) and high-throughput RNA sequencing (RNA-seq). We then identified a large number of differentially methylated regions (DMRs) in the comparison of major psychosis and normal subjects. These DMRs are widely distributed in different functional elements, such as promoters, CpG islands (CGIs), non-coding RNAs (e.g., miRNAs and lncRNAs) and repetitive elements, especially in introns. We found that about fifty percent of intronic DMRs seemed to affect transcription elongation. Further, we observed enhancer-related DMRs (such as p300 binding in hypermethylated DMRs) that could impact TF binding, therefore affecting gene expression. In particular, hypomethylated DMRs were found to be significantly enriched for Pol3 binding. In addition, by functional enrichment analysis for differential targets of hypermethylated or hypomethylated intronic miRNAs, we found that DNA methylation aberrations of intronic miRNAs could better account for functional alterations in major psychosis. Collectively, we demonstrated that DNA methylation alterations might influence gene expression in an indirect manner through intronic miRNAs.

## Methods

### Ethics statement

We obtained ethics approval for our study from Southwest Brain Bank with consent from the next-of-kin (NOK) (see Supplemental Methods for details in Additional file 1). The NOKs agreed to provide the donation and they read a State approved form. The NOKs provided verbal consent prior to their telephone interview. We called the NOKs and recorded their agreement. All NOKs provided informed consent and explicitly agreed.

### Patient samples

Five SZ, seven BD and six normal samples were included in this study, which were collected from the Southwest Brain Bank with consent from the next-of-kin (NOK). The NOK interview (psychological autopsy) about the donor was performed by trained clinicians. All of the patients in this study have met best estimate consensus diagnoses of SZ or BD as defined by the DSM-IV-TR criteria, as previously reported [24]. These studies have been approved by the Institutional Review Board of the University of Texas Health Science Center at San Antonio. The quality of the postmortem brain tissue (BA9) was determined by a neuro-pathologist through both gross and microscopic neuropathological examinations. All subjects in this study were free of confounding neuropathology. For tissue identification of the brain region BA9 taken from the same hemisphere, we used the criteria described by Rajkowska and Goldman-Rakic [25]. Detailed sample descriptions are shown in Additional file 1: Table S1.

### MeDIP-seq

We sonicated genomic DNA to produce random fragments with an average size of approximately 250 bp. Sonicated DNA was then end-repaired, A-tailed and ligated with sequencing adapters following the standard Illumina library preparation protocol. Double-stranded DNA was denatured and immunoprecipitated with 5-methylcytidine antibody. The immunoprecipitated DNA was amplified by PCR and the efficiency of immunoprecipitation was validated by real-time PCR. Genome-wide massively parallel paired-end sequencing was subsequently performed on the Illumina HiSeq 2000 platform according to the manufacturer’s instructions. The MeDIP-seq data from this study have been submitted to NCBI Sequence Read Archive under accession no. SRP035524.

### RNA-seq

Beads with oligo(dT) were used to isolate poly(A) mRNA after total RNA was collected from all samples (RNeasy Lipid Tissue Mini Kit (Qiagen # 74804) and QIAzol Lysis Reagent (Qiagen # 79306)). In the study, only high-quality RNA samples that yielded RNA integrity numbers (RIN) scores > = 6.0 by Agilent Bioanalyzer were used for subsequent analysis. The average RIN scores for SZ, BD and control samples were 7.6, 7.3 and 7.4, respectively. And there were no significant differences of RIN scores between disease (SZ or BD) and control samples (*P* value =0.81 and 0.67, respectively, two-tailed Student *t* test). mRNA was fragmented in fragmentation buffer as previously described [26]. Using these short fragments as templates, the first-strand cDNA was synthesized with random hexamer-primer (TaqMan Gene Expression Assays). The buffer, dNTPs, RNase H and DNA polymerase I were used to synthesize the second-strand cDNA. QiaQuick PCR extraction kit was used to purify short fragments. These short fragments were subsequently resolved with EB buffer for end reparation and adding poly(A) and then connected with sequencing adaptors. Based on the results of agarose gel electrophoresis, PCR amplification was done by selecting suitable fragments as templates. The library was then sequenced with 90 bp paired-end reads on Illumina HiSeq 2000. The RNA-seq data from this study have been submitted to NCBI Sequence Read Archive under accession no. SRP035524.

### Genomic elements

The genomic coordinates of CpG islands (CGIs), exons, introns, 5′UTR and 3′UTR were obtained from UCSC genome browser [27]. Promoters were defined as 2500 bp upstream and 500 bp downstream of the transcriptional start sites (TSSs). CGI shores were defined as 2 kb from CGI edge. Noncoding RNAs were downloaded from GENCODE v.10 [28]. We obtained genome-wide functional elements which were characterized by multiple epigenetic marks using a Hidden Markov Model [29] from UCSC genome browser, such as strong or weak enhancers and transcriptional elongation.

### miRNAs and their targets

The genomic coordinates of human miRNAs were retrieved from miRBase (release 20) [30]. Human miRNA TSSs were downloaded from miRStart (http://mirstart.mbc.nctu.edu.tw/) [31] and their promoter regions were defined as 2500 bp upstream and 500 bp downstream of TSS. Then we downloaded miRNA-target relations from TargetScan (http://www.targetscan.org/) [32] with a context score ≤ −0.3.

### Identification of differentially methylated regions

High-quality MeDIP-seq reads were aligned against the human genome (hg19) using SOAP2 (version 2.20) [33]. Only the uniquely mapped reads were used for further analysis. These reads were analyzed using the MEDIPS software (version 1.10.0) [34] to quantify relative DNA methylation levels in sliding window of 50 bp. Differential methylation analysis was carried out using MEDIPS (FDR < 0.05). Differentially methylated windows with a distance less than 1000 bp were then merged. Regions longer than 500 bp with at least 50 % of 50 bp windows showing significant methylation difference were regarded as differentially methylated regions (DMRs).

### Identification of differentially expressed genes

After removing low-quality reads (reads containing Ns > 5), the remaining reads were mapped to human reference genome (hg19) using SOAP2. Mismatches of no more than 2 bases were allowed in the alignment. Transcripts were assembled using Cufflinks 2.1.1 with default parameters as proposed by Trapnell et al. [35]. Gene expression values were estimated as FPKM values using Cufflinks. Cuffdiff [35] was used for discovery of the differentially expressed genes (FDR < 0.05).

### Permutation test

Permutation tests were performed to assess statistical significance of the enrichment of DMRs in specific type of genomic elements (such as promoters and introns). As for each type of genomic element, the same amount of genomic regions were randomly selected from genomes, maintaining the same distribution of chromosome and length as real DMRs. This process was then repeated 1000 times to generate 1000 random DMR sets. The observed value was defined as the real number of DMRs overlapping with these regions. And the average number of DMRs in random sets showing overlap with these regions was regarded as the expected value. Then the observed-expected (O/E) ratio of DMRs was calculated. The *P* value was computed as the percentage of random DMR sets that showed more overlap with these regions than the real DMR set.

### Functional enrichment analysis

We calculated GO term enrichments (biological processes) using the hypergeometric test with FDR < 0.05 (GOstats package in Bioconductor).

## Results

### Identification of differentially methylated regions (DMRs)

To characterize the global DNA methylation alterations of major psychosis, we determined high-resolution methylation maps of brain samples from 5 SZ, 7 BD and 6 normal subjects using MeDIP-seq. Paired-end reads were aligned to the human reference genome (NCBI Build 37) and the saturation analysis indicated that the sequencing depth is sufficient to analyze genome-wide DNA methylation patterns (Additional file 1: Figure S1). Then, compared with normal subjects, we identified 10,961 DMRs (7880 hypermethylated and 3081 hypomethylated) in SZ patients and 16,599 DMRs (6836 hypermethylated and 9763 hypomethylated) in BD patients.

**Fig. 1:**
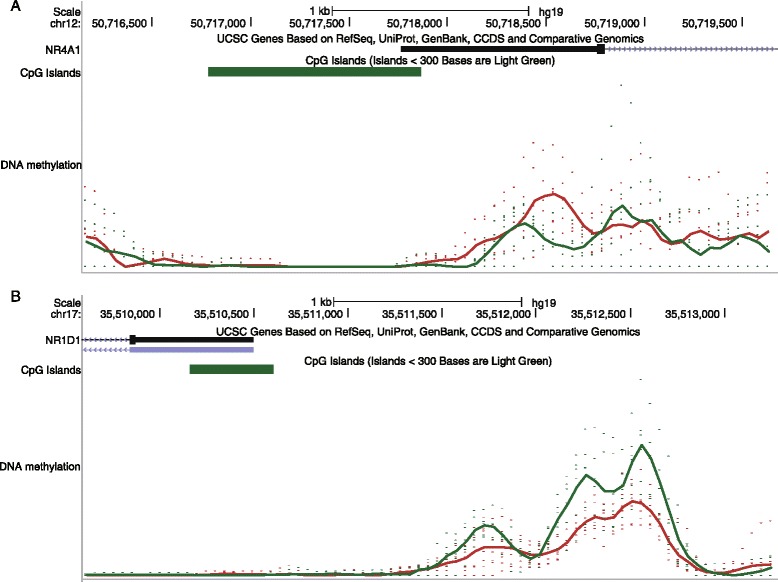
The screenshots of aberrant DNA methylation for NR4A1 in SZ (**a**) and NR1D1 in BD (**b**), respectively. The *curves* represent the average DNA methylation levels (*red* for disease and *green* for normal samples). CpG islands are shown by *green rectangle*

Considering that pathology of many diseases are closely related to aberrant DNA methylation, especially at gene promoters. We therefore focused on the effect of aberrant DNA methylation at gene promoters on gene expression. We found that many DMRs tended to overlap with the promoters of known SZ- and BD-related genes (Table 1). For example, NR4A1, whose promoter overlaps with DMRs, has been implicated in several diseases, such as SZ, Alzheimer’s disease as well as cancer [36]. Significantly lower mRNA expression levels and protein abundance of NR4A1 were observed in SZ patients when compared with controls [37]. In line with these findings, we observed a hypermethylated DMR at the promoter of NR4A1 (Fig. 1a), which might interpret the lower expression to some extent. Another one, NR1D1, has been repeatedly demonstrated to be linked with BD [38]. In agreement with previous studies, our results showed that the upstream region of its promoter exhibited an obvious hypomethylation pattern (Fig. 1b).

**Table 1:** Disease-related gene list with aberrant methylation supported by literature in SZ and BD, separately

Phenotype	Known genes with aberrant methylation
SZ	PLP1, NR4A1, IL1B, GFAP, APC, TAAR1, MYT1L, GRIP1, ASTN2, EGFR, CD28 and SLC6A2
BD	DNMT1, NR1D1, PDLIM5, GABRR1, GABRA4, CACNA1C, GCLM, NDEL1, NTRK1, ABCA13, IGF1, BCR, PPP3CC, KIF17, OPRM1, ACSL6, SREBF1, RUNX2, SOD2, PRDX6, PLXNA2, RAI1, MYT1L and ATM

### Characterization of genome-wide distributions of DMRs and their associations with expression changes

**Fig. 2:**
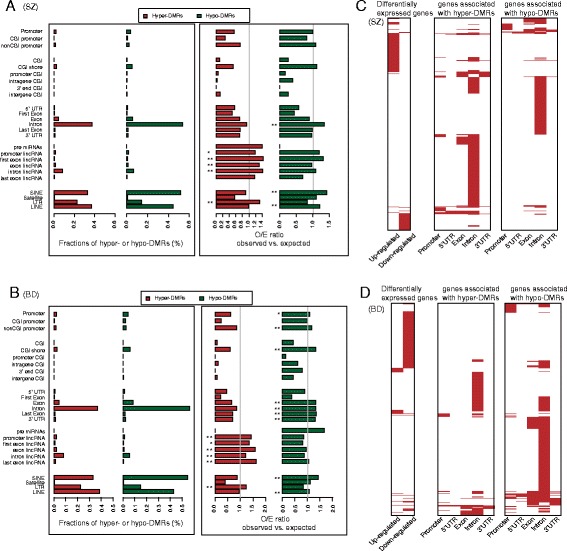
Distribution of hyper-DMRs or hypo-DMRs in different genomic elements. **a**, **b** Left panel: Each bar represents the fraction of hyper-DMRs (or hypo-DMRs) map to each genomic element in SZ (**a**) and BD (**b**). Right panel: The x axis indicates O/E ratios between the observed and expected number of DMRs overlapping with a given type of genomic elements. The average number of DMRs in random sets overlapping with a given type of genomic elements was regarded as the expected value. Asterisks indicating the significance levels (* represents *P* < 0.05; ** represents *P* < 0.01). **c**, **d** Comparisons between differentially expressed genes and genes with aberrant DNA methylation in different genomic elements in SZ (**c**) and BD (**d**). Distribution of up- and down-regulated genes (*left*), and genes with hyper-DMRs (*middle*) or hypo-DMRs (*right*) located in their different genomic elements. For each type of genes, *red* indicates present

In order to analyze the distribution of DMRs across whole genome, we retrieved different genomic elements, such as CGIs, promoters, exons and introns (see Methods). Then we found that a high percent of DMRs fell into introns and repeat elements (such as, SINE and LINE), in both SZ and BD (Fig. 2). By randomly generating the same number of DMRs, our results demonstrated that hypermethylated DMRs significantly overlapped with long intergenic non-coding RNAs (lincRNAs) and LTR in SZ (*P* < 0.05, permutation test; Fig. 2a, right panel). This is supported by recent discoveries that aberrantly epigenetic- modified lincRNAs contributing to their expression changes function in important cellular processes [39]. In contrast, hypomethylated DMRs significantly overlapped with introns, SINE and LINE. Similar enrichment patterns across different genomic elements were also observed for hypermethylated and hypomethylated DMRs in BD (Fig. 2b, right panel). These results suggested an inherent property of DNA methylation alterations across different genomic elements in major psychosis.

To further examine the effect of DMRs on gene expression, we analyzed the transcriptomes of major psychosis using RNA-seq. There are 1077 and 2085 differentially expressed genes in SZ and BD, respectively (see Methods). In addition, we extracted DMR-related genes and grouped them into different element-associated sets. By comparing these genes with differentially expressed genes, we found that there are 288 genes for SZ and 557 genes for BD with aberrant promoter methylation, of which 14 (4.9 %) and 73 (13.1 %) showed expression changes (Fig. 2c and Additional file 1: Table S2 for SZ; Fig. 2d and Additional file 1: Table S3 for BD). It may lead us to consider that DNA methylation had a limited role in directly regulating gene expression. Interestingly, there were 3232 genes for SZ and 5107 genes for BD with intronic DMRs, of which 236 (7.3 %) and 499 (9.8 %) showed expression changes (Additional file 1: Table S2 for SZ; Additional file 1: Table S3 for BD). These findings suggested that beside the impact of promoter DNA methylation on gene expression, DNA methylation alterations in introns might represent another mechanism by which DNA methylation influences expression.

### Enrichment of DMRs in introns affecting diverse functional elements

**Fig. 3:**
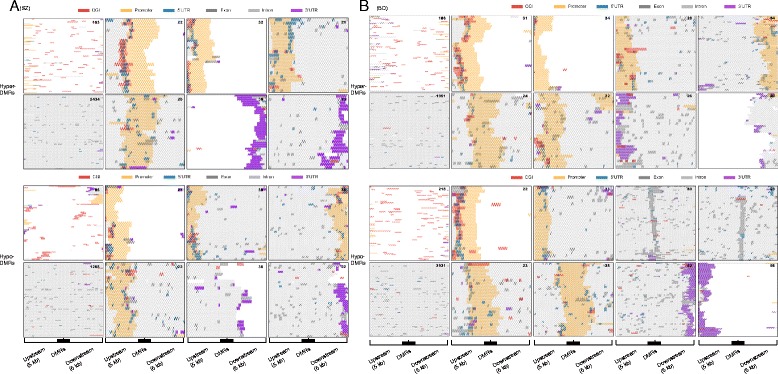
The distribution patterns of genomic elements around DMRs in SZ (**a**) and BD (**b**). Upper and lower panels represent hypermethylated and hypomethylated DMRs, respectively. Each hyper- or hypo-DMR was divided into 10 equal-sized intervals. The regions 5 kb upstream and downstream of the DMR were divided into 200-bp intervals. Each heat map presents a distribution pattern of different genomic elements around hyper-DMRs or hypo-DMRs. Intervals overlapping with different genomic elements were indicated by different colors

Next, we sought to examine how different genomic elements are distributed around DMRs. We analyzed the distribution patterns of different genomic elements (such as CGI, promoter and intron) around hypermethylated or hypomethylated DMRs in SZ and BD. Figure 3 shows diverse distribution patterns. Some DMRs directly overlap with promoters and some are located on their flanks. However, many DMRs (2434 hypermethylated and 1268 hypomethylated DMRs in SZ; 1951 hypermethylated and 3931 hypomethylated DMRs in BD) completely fell into introns, although a few were mapped to promoters (Fig. 3). Therefore, we investigated whether these intronic DMRs affected different types of functional elements defined by epigenetic marks, which in turn contributed to gene expression changes.

**Fig. 4:**
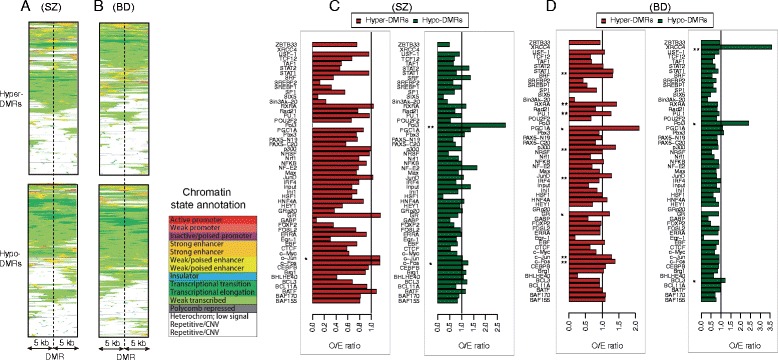
Relationship between intronic DMRs and functional elements. **a**, **b** Heatmaps showing different types of functional elements defined by multiple epigenetic marks at or around hypermethylated and hypomethylated intronic DMRs in SZ (**a**) and BD (**b**). **c**, **d** Enrichment of hypermethylated and hypomethylated intronic DMRs in transcription factor binding. Each bar represents the O/E ratio between the observed and expected number of intronic DMRs overlapping with the TF binding sites in SZ (**c**) and BD (**d**). Asterisks indicating the significance levels (* represents *P* < 0.05; ** represents *P* < 0.01)

We used a multivariate hidden Markov model [29] to determine 15 different functional chromatin states, which represented different functional elements (such as, insulators and enhancers), based on combinations of different epigenetic marks in human embryonic stem cells. Subsequently, we analyzed the distribution of different functional elements across the 10 kb regions surrounding intronic DMRs in SZ and BD. Although a part of intronic DMRs are marked by polycomb repressed, heterochromatic and repetitive states, more than 50 % of the intronic hyper- and hypomethylated regions in SZ and BD are associated with either strongly or weakly transcribed regions (Fig. 4a and b). Such association between intronic DMRs and transcriptional activity supported an important role of intronic DNA methylation in altering chromatin structure and elongation efficiency [40]. Note that it is not necessarily true that hypermethylation and hypomethylation are correlated with regression and activation of transcription, respectively. In addition, we also found that a few intronic DMRs were associated with active and weak promoters (Fig. 4, red) and more with strong and weak enhancers (Fig. 4, yellow), implying that intronic DMRs were involved in gene regulation likely by interfering the long-range looping interactions between promoters and enhancers.

DNA methylation can also contribute to gene transcription through changing chromatin structure and, in turn, influence the affinity and accessibility of transcription-factor binding sites (TFBS) [41]. Thus, we wanted to know whether intronic DMRs were associated with transcription-factor binding. To address this issue, we detected genome-wide binding sites for each transcription factor by MACS (version 1.4.2, *P* < 10^−5^) using ChIP-seq data for 55 human transcription factors from ENCODE project. For each TF, we calculated the number of intronic DMRs overlapping with TFBSs, and then performed permutation tests to assess statistical significance (Fig. 4c, d).

We found that aberrant DNA methylation disrupts a lot of TFBSs in introns. For example, intronic DMRs were significantly enriched in the binding sites of the transcription complex *c-Jun* and *c-Fos* in both SZ and BD (Fig. 4c, d). *c-Fos* has been implicated in the control of genetic events in neurons, which can dimerize with proteins of the *JUN* family, thereby forming the transcription factor complex AP-1. AP-1 can engage in neurodegeneration and neuroregeneration by regulating brain gene expression, such as *TNF-alpha*, *Bcl-3* and *MCP-1* [42]. Intronic hypermethylated DMRs in BD disrupts binding sites for enhancer-associated protein p300 [43], highlighting the tight associations of intronic DMRs with enhancers. In BD, we observed a significant enrichment of intronic hypomethylation on binding sites of *Bcl3*. This gene encodes a protein functioning as a transcriptional co-activator, through interacting with NF-kappa B (NF-κB) homodimers. As proposed by Kaltschmidt et al. [44], NF-κB was abundant in neurons and played a role in neurological disorders.

It should be noted that even though the majority of intronic DMRs can interfere with transcriptional initiation, elongation or enhancer-meditated long-range looping interactions, the discrepancy between DNA methylation and gene expression is still difficult to explain.

### DMR-mediated dysfunction of intronic miRNAs contributing to expression changes

Interestingly, we found that intronic DMRs showed significant overlap with the binding sites of RNA Polymerase III (Pol3) in both SZ and BD (Fig. 4c, d). As we know, Pol3 can transcribe different types of noncoding RNAs [45], such as miRNAs [46]. Given the fact that the majority of human miRNAs are located within the intronic regions of transcription units [47], we therefore suspected that intronic DMRs may influence miRNA transcription.

**Fig. 5:**
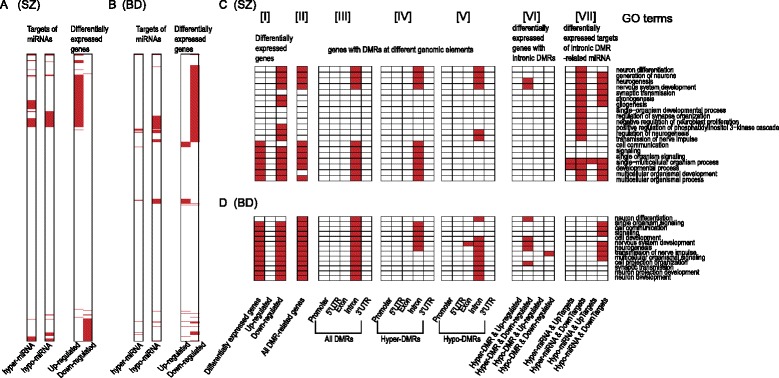
DMRs-mediated dysfunction of intronic miRNAs contributing to expression changes. **a**, **b** Distribution of targets of hypermethylated or hypomethylated intronic DMR-related miRNAs (i.e. hyper-miRNA or hypo-miRNA) and differentially expressed genes in SZ (**a**) and BD (**b**). **c**, **d** Heat maps of GO terms enriched by different gene sets: [I] differentially expressed genes, up- and down-regulated genes; [II] DMR-related genes; [III-V] genes with DMRs, hyper-DMRs and hypo-DMRs at different genomic elements (including promoter, 5′UTR, exon, intron and 3′UTR); [VI] hypermethylated or hypomethylated intronic DMR-related genes with expression changes; [VII] up- or down-regulated targets of hypermethylated or hypomethylated intronic DMR-related miRNAs, in SZ (**c**) and BD (**d**)

In order to investigate whether aberrant DNA methylation-associated intronic miRNAs account for gene differential expression, we obtained intronic miRNAs whose promoters are covered by DMRs and retrieved their targets from TargetScan. The results showed that targets of intronic DMR-related miRNAs captured numerous differentially expressed genes in SZ and BD (Fig. 5a, b). It suggested that a part of dysregulated genes might be mediated by aberrant DNA methylation of intronic miRNAs.

For example, in SZ, *hsa-mir-7-3* was identified as a hypomethylated intronic miRNA, and its increased expression has been validated in previous studies [48]. Other hypomethylated intronic miRNAs, such as *hsa-mir-128-1* [49], *hsa-mir-590* [50] and *hsa-mir-455* [51], have also been reported to be involved in brain-related diseases. Together, these results demonstrated that aberrant DNA methylation could induce dysfunction of intronic miRNAs, and in turn change expressions of their targets, suggesting an indirect effect of methylation on expression changes in major psychosis.

### Functional characterization of intronic DMR-related miRNAs

To investigate whether these intronic DMR-related miRNAs are responsible for important functions in major psychosis, we first performed functional enrichment analysis based on all differentially expressed genes, up-regulated and down-regulated genes separately (Fig. 5c-[I] for SC, Fig. 5d-[I] for BD). These differentially expressed genes were involved in many important biological processes, such as neurogenesis, consistent with previous studies. To our surprise, up-regulated genes are not significantly enriched for any biological functions. In contrast, down-regulated genes can capture many important functions associated with major psychosis, such as neurogenesis and nervous system development. These findings might suggest that down-regulated genes may play more important roles in major psychosis relative to up-regulated genes.

Subsequently, we sought to use DMR-related genes to capture biological functions characterized by differentially expressed genes. We found that only the intronic DMR-related genes can accurately cover the majority of these functions in both SZ and BD (Fig. 5c-[III-V] and Fig. 5d-[III-V]), while DMRs falling into other elements (including promoter, 5′UTR, exon and 3′UTR) almost cannot capture any functions. Unexpectedly, when combining DNA methylation alterations with gene expression changes, only down-regulated genes with hypermethylated intronic DMRs can capture limited functions enriched by differentially expressed genes in both SZ and BD (Fig. 5c-[VI] and Fig. 5d-[VI]). It thus inspires us to wonder whether intronic DMR-related miRNAs could contribute to these abnormal functions in major psychosis. To address this issue, we examined the functions of up- or down-regulated targets of intronic DMR-related miRNAs. In both SZ and BD, we observed that the down-regulated targets of hypomethylated intronic miRNAs could effectively account for the functions enriched by differentially expressed genes (Fig. 5c-[VII] and Fig. 5d-[VII]). For example, in SZ, 74 down-regulated genes, targeted by 11 hypomethylated intronic miRNAs, were found to be significantly enriched in neurogenesis. These findings strongly indicated that intronic DMR-related miRNAs play important functions by disturbing their targets in major psychosis.

## Discussion

In this study, we described an integrative DNA methylome and transcriptome analysis of major psychosis concerning SZ and BD by using MeDIP-seq and RNA-seq, and explored how the epigenetic dimensions influenced expression and in turn contributed to the abnormal functions. Firstly, we identified a large number of DMRs that were distributed across different genomic elements and epigenetic-modified functional elements. By comparing DMR-related genes with differentially expressed genes, we found that DNA methylation alterations can only account for limited gene expression changes. Such discrepancy between DNA methylation and gene expression probably suggests their indirect effects. We further observed that many intronic DMRs overlapped with miRNAs. Functional analysis results showed a possible role of miRNAs involved in DMR-induced gene expression changes.

Consistent with previous studies, DNA methylation alternations at gene promoters could regulate a part of gene expression (Additional file 1: Table S4 for SZ and S5 for BD). For SZ, we found that 4 of the 168 genes with promoter hypermethylation showed decreased expression and 6 of 120 genes with promoter hypomethylation showed increased expression (Additional file 1: Table S4). For BD, 11 of the 146 genes with promoter hypermethylation showed decreased expression and 4 of 411 genes with promoter hypomethylation showed increased expression (Additional file 1: Table S5). As an example, gene *PLP1* gained increased promoter methylation levels and showed down-regulated expression in SZ patients compared with control subjects (Additional file 1: Figure S2). Decreased expression of *PLP1* has been frequently observed in BA9 of patients with both SZ and BD [52]. Interestingly, we found that many DMRs were located in introns, probably affecting promoters, transcriptional elongation and enhancer-mediated looping, in line with a recent report that intragenic methylation could play a major role in the regulation of tissue- and cell-specific alternative promoters [53]. Interestingly, we found that a high percent of intronic DMR overlapped with miRNAs. Also, we found that many target genes of the aberrant DNA methylation-related miRNAs were differentially expressed, suggesting that DNA methylation alteration might indirectly affect global gene expression by miRNAs. To further determine whether differential targets of DMR-related miRNAs are responsible for psychosis-related biological processes, we performed functional comparison analysis. Our results showed that intronic DMR-related miRNAs were involved in many important biological processes in major psychosis. These DMR-related miRNAs could more extensively explain the abnormal functions than protein-coding genes directly affected by DMRs. Indeed, previous studies have reported that dysregulation of miRNAs was frequently observed in major psychosis [16, 54]. Beveridge et al. [51] demonstrated a significant expression increase of global miRNAs in SZ. The global expression increase probably resulted from aberrant DNA methylation, which is supported by a recent report that widespread hypomethylation was observed in frontal cortex of both SZ and BD subjects [12]. Hence, dysregulated gene expression might not be directly caused by aberrant methylation, but indirectly mediated by dysregulated miRNAs.

Given that promoter methylation could account for a part of expression changes of protein coding genes (PCGs), it remains to be determined whether these finding is applicable to miRNAs. To address this, we downloaded an additional set of RNA-seq, smRNA-seq and MeDIP-seq data of H1 cell line from the NIH Roadmap Epigenomics Program [55] and calculated the expression levels of miRNAs and PCGs as well as their promoter methylation levels. As a result, both miRNAs and PCGs showed negative correlations between promoter methylation and their expression (Pearson’s correlation coefficient = −0.08, *P* value < 2.2e^−16^ for PCGs and Pearson’s correlation coefficient = −0.08, *P* value = 0.1 for miRNAs; Additional file 1: Figure S3). These results suggested that there were no significant differences in the effects of DNA methylation on expression of PCGs and miRNAs.

Long intergenic noncoding RNAs are key regulators of chromatin states for important biological processes, contributing to the development and progression of diverse diseases [56]. They can silence or activate genes by guiding chromatin remodeling complexes to specific target genes, such as recruiting repressive complexes such as PRC2 or the DNA demethylation machinery to promoters. Interestingly, in our results, we observed that hypermethylated DMRs were significantly enriched for lincRNAs in both SZ and BD (Fig. 2a, b). The enrichment of hypermethylated DMRs in lincRNAs indicated that DNA methylation alteration might influence expression of lincRNAs. Like miRNAs, these DMR-related lincRNAs could further regulate expression of downstream genes, which may represent another indirect mechanism for explaining the relationship between DNA methylation and gene expression. Thus, exploring how aberrant DNA methylation affects the specific functions of lncRNAs in major psychosis may provide novel insights into the underlying molecular mechanisms.

## Conclusions

In conclusion, we detected an interesting link between DNA methylation and gene expression in major psychosis by integrating epigenome and transcriptome. Our results showed that DNA methylation would influence gene transcription not only by aberrant promoter methylation directly but also by miRNA-related intronic methylation changes indirectly. Characterization of dysfunctional miRNAs mediated by aberrant DNA methylation will facilitate understanding of the pathogenesis of major psychosis.

### Availability of supporting data

All supporting data are included as additional files or kept in publicly available repositories. The MeDIP-seq and RNA-seq data from this study have been submitted to NCBI Sequence Read Archive under accession no. SRP035524 (http://trace.ncbi.nlm.nih.gov/Traces/sra/?study=SRP035524). The genomic coordinates of CpG islands (CGIs), exons, introns, 5′UTR and 3′UTR were obtained from UCSC genome browser (http://genome.ucsc.edu/). Human miRNA TSSs are freely available at http://mirstart.mbc.nctu.edu.tw/. The miRNA-target relations used in this article are freely available in the TargetScan database (http://www.targetscan.org/).

## Additional file

Additional file 1:
**Supplemental Methods.** Subjects. Postmortem tissue. DNA and RNA preparation. **Figure S1.** Saturation analysis. Analysis was performed for each sample, with the number of reads plotted on the x-axis and correlation coefficient on the y-axis. **Figure S2.** The UCSC Browser screenshot showing raw RNA-seq and MeDIP-seq reads distributions around gene *PLP1* in SZ patients and controls. Yellow shadows represent the regions with differential expression (up) and methylation (bottom), respectively. **Figure S3.** Scatter diagrams showing the correlations between promoter methylation levels (x axis) and log-transformed expression (y axis) of protein coding genes (left) and miRNAs (right). **Table S1.** Demographic characteristics for 18 subjects**. Table S2.** The number (percentage) of genes with hyper vs hypomethylated elements and gene expression (up or down) for SZ. **Table S3.** The number (percentage) of genes with hyper vs hypomethylated elements and gene expression (up or down) for BD. **Table S4.** The number of genes with hyper vs hypomethylated promoters and gene expression (up or down) for SZ. **Table S5.** The number of genes with hyper vs hypomethylated promoters and gene expression (up or down) for BD. (DOC 21 kb)

## Abbreviations

DMRs: Differential DNA methylated regions
SZ: Schizophrenia
BD: Bipolar disorder
miRNAs: microRNAs
TFs: Transcription factors
MeDIP-seq: Methylated DNA immunoprecipitation and sequencing
RNA-seq: High-throughput RNA sequencing
CGIs: CpG islands
lincRNAs: Long intergenic non-coding RNAs
TFBS: Transcription-factor binding sites
NF-κB: NF-kappa B
Pol3: RNA Polymerase III
NOK: Next-of-kin
TSSs: Transcriptional start sites
PCGs: Protein coding genes
