# Supplementary material for: Genome-wide DNA methylome reveals the dysfunction of intronic microRNAs in major psychosis
Source: BMC Med Genomics. 2015 Oct 14;8:62. doi: 10.1186/s12920-015-0139-4 (PMC4604612; doi:10.1186/s12920-015-0139-4)
Supplement: Additional file 1: — Supplemental Methods. Subjects. Postmortem tissue. DNA and RNA preparation. Figure S1. Saturation analysis. Analysis was performed for each sample, with the number of reads plotted on the x-axis and correlation coefficient on the y-axis. Figure S2. The UCSC Browser screenshot showing raw RNA-seq and MeDIP-seq reads distributions around gene PLP1 in SZ patients and controls. Yellow shadows represent the regions with differential expression (up) and methylation (bottom), respectively. Figure S3. Scatter diagrams showing the correlations between promoter methylation levels (x axis) and log-transformed expression (y axis) of protein coding genes (left) and miRNAs (right). Table S1. Demographic characteristics for 18 subjects. Table S2. The number (percentage) of genes with hyper vs hypomethylated elements and gene expression (up or down) for SZ. Table S3. The number (percentage) of genes with hyper vs hypomethylated elements and gene expression (up or down) for BD. Table S4. The number of genes with hyper vs hypomethylated promoters and gene expression (up or down) for SZ. Table S5. The number of genes with hyper vs hypomethylated promoters and gene expression (up or down) for BD. (DOC 21 kb) [file 12920_2015_139_MOESM1_ESM.doc]

Supplemental Methods


Subjects


Brain tissues from control [1], schizophrenia (N=5, SC) and bipolar disorder (N=7, BP) subjects were obtained from the Southwest Brain Bank (SWBB). The SWBB collection of postmortem tissue for research was conducted under the jurisdiction of the State of Texas Anatomical Review Board. All interviews with the NOK have been determined to be exempt from ethical review by the The University of Texas Health Science Center at San Antonio (UTHSCSA) IRB. The NOK agreed to provide the donation and they read a State approved form. We telephoned the NOK and recorded their agreement to donate. The NOK interview (psychological autopsy) about the donor  was  performed  by  trained  clinicians.  To  establish  the  clinical  diagnosis  a DSM-IV  based  Mini-International  Neuropsychiatric  Interview  (M.I.N.I.)[2]  was administrated to the NOK about the deceased. This information in addition to all medical  records  that  were  obtained  was  presented  to  the  expert  diagnostician consensus group, whose inter-rater reliability for the MINI was 0.8 for SC and BP. Medications listed were those that were prescribed at the time of death. In order to estimate  psychotic  symptoms  in  the  subjects  at  the  time  of  death  in  the  NOK interview  a  retrospective  Bipolar  Inventory  of  Symptoms  Scale  (BISS)  was administrated. The BISS generated a total severity score and 5 symptom factor scores: mania,  depression,  anxiety,  irritability  and  psychosis[3].  The  psychosis  factor composed of the questions about paranoid delusions, hallucinations and impaired insight was used to estimate the presence of psychosis in the last week of life.

Postmortem tissue


The cerebrum was hemisected and cut into 1 cm-thick coronal blocks starting at the frontal pole, digitally photographed to document anatomical location, immediately frozen in isopentane (2-methylbutane, Fisher), chilled with dry ice to -60°C, and then stored at -80°C. Tissue quality was determined by a neuro-pathologist through both gross and microscopic neuropathological examinations. All subjects included in this study were free of confounding neuropathology. Toxicology results were obtained from the Bexar County Forensic Toxicology Laboratory via the autopsy report. Control tissue was toxicology free of neurotropic medications (Table S1).
DNA and RNA preparation


All of our samples were from fresh-frozen specimens that were stored in -80   C freezers.    Tissue storage time was 1-3 years. The samples were collected from the larger blocks containing the appropriate brain region and RNA/DNA was isolated within a few days of dissection from the larger blocks of tissue. These samples were from postmortem cases with SC and BP who had a postmortem interval (PMI) (the time of death until the time of tissue preservation) of about 24 hours. As a result, RNA integrity (RIN) was lower compared to animal models or in vitro studies.

Figure Sl


--1

I· I. I

----


;=-t

j, L I

---
;= -t

I· I. I


-,f·t.­__...


- 1 1 =--=-t

t • I I

---..·...-....

;<:::: 1

l. I
I
'1 1:-=:- 1

I· I
I

·- ·-
--·'-"'--'
11 =;=-t


.....
-11 =--=-t

I·
I
I

---·.-,··--·-


I· L I


---
IL=  -J

t
L
I

---.-..·.·--..-..


;=-_t

I.
I
I


_,.,.- , ·,_­,.,.-..,,
----


- ·-.·,·-l  , -


---·..·..·--··
;;,::::-    • I

I·	I·
I·	l·


: ;z::;

I·
L
J


-·-·-··-·-··
.t

I·
L
l


---·-"·'·---··
- I

I· I. l


--·-··--·--··

Figure S1. Saturation analysis. Analysis was performed for each sample, with the number of reads plotted on the x-axis, and correlation coefficient on the y-axis.


Figure S2


Figure S2. The UCSC Browser showing raw RNA-seq and MeDIP-seq reads distributions  around  gene  PLP1  in  SZ  patients  and  controls.  Yellow  shadow represents the DNA methylation levels in the promoter region of PLP1.

Figure S3


Figure S3. Scatter diagrams showing the correlations between promoter methylation levels (x axis) and log-transformed expression (y axis) of protein coding genes (left) and miRNAs (right).


Table S1. Demographic characteristics for 18 subjects.


Subject ID	Group	PMI	race	age at onset
comorbid alcohol
dependence

psych meds

Antidepressant meds


benzodiazepine
meds	Neuroleptic

mood stabilizers


13	BP	13	h	NA	0	0	0	0	0	0	
37	BP	29	h	31	1	1	1	0	0	0	
67	BP	33	w	NA	0	0	0	0	0	0	
78	BP	31	w	27	0	1	1	0	0	0	
91	BP	32	w	20	1	1	1	1	0	1	
96	BP	27	w	29	1	1	1	0	0	0	
114	BP	24	w	13	1	1	1	0	0	0	
79	Control	26	w	NA	0	0	0	0	0	0	
108	Control	20	w	NA	0	0	0	0	0	0	
122	Control	29	h	NA	0	0	0	0	0	0	
130	Control	23	w	NA	0	0	0	0	0	0	
144	Control	18	w	NA	0	0	0	0	0	0	
162	Control	31	w	NA	0	0	0	0	0	0	
62	SC	36	h	36	1	0	0	0	0	0	
84	SC	23	w	25	0	0	0	0	0	0	
106	SC	29	w	17	0	1	1	1	1	0	
107	SC	26	w	35	0	1	1	1	0	0	
152	SC	36	h	19	0	1	0	0	1	0	
PMI =Post-mortem Interval; race, h = Hispanic population, w = whilte population, NA = not avaiable


Table  S2.  The  number  (percentage)  of  genes  with  hypermethylated  or  hypomethylated  regions  in  different  elements  showing  up-  or down-regulated expression in SZ.


Element	
promoter	
5’ UTR	
exon	
intron	
3’ UTR	

Expression change	
up	
down	
up	
down	
up	
down	
up	
down	
up	
down	

hypermethylated	
4 (1.4%)	
4 (1.4%)	
5 (6.0%)	
2 (2.4%)	
26 (5.0%)	
5 (1.0%)	
122 (3.8%)	
41 (1.3%)	
6 (4.8%)	
1 (0.8%)	

hypomethylated	
6 (2.1%)	
0	
0	
1 (1.2%)	
6 (1.2%)	
2 (0.4%)	
63 (1.9%)	
10 (0.3%)	
1 (0.8%)	
0	


Table  S3.  The  number  (percentage)  of  genes  with  hypermethylated  or  hypomethylated  regions  in  different  elements  showing  up-  or down-regulated expression in BD.


Element	
promoter	
5’ UTR	
exon	
intron	
3’ UTR	

Expression change	
up	
down	
up	
down	
up	
down	
up	
down	
up	
down	

hypermethylated	
2 (0.4%)	
11 (2.0%)	
1 (0.6%)	
2 (1.2%)	
2 (0.2%)	
26 (2.4%)	
28 (0.5%)	
138 (2.7%)	
0	
3 (1.1%)	

hypomethylated	
4 (0.7%)	
56 (10.1%)	
1 (0.6%)	
15 (9.4%)	
8 (0.7%)	
94 (8.6%)	
30 (0.6%)	
303 (5.9%)	
2 (0.8%)	
23 (8.7%)	

Table S4. The number of genes with hypermethylated or hypomethylated promoters and gene expression (up or down) for SZ.


	Up-regulated (761)	Down-regulated (316)	
Hypermethylation (168)	4	4	
Hypomethylation (120)	6	0	


Table S5. The number of genes with hypermethylated or hypomethylated promoters and gene expression (up or down) for BD.


	Up-regulated (230)	Down-regulated (1855)	
Hypermethylation (146)	2	11	
Hypomethylation (411)	4	56	


References


1. Del Vecchio A, Latini G, Henry E, Christensen RD. Template bleeding times of 240 neonates born at
24 to 41 weeks gestation. J Perinatol. 2008;28(6):427-31. doi:10.1038/jp.2008.10 jp200810 [pii].
2. Sheehan DV, Lecrubier Y, Sheehan KH, Amorim P, Janavs J, Weiller E et al. The Mini-International Neuropsychiatric Interview (M.I.N.I.): the development and validation of a structured diagnostic psychiatric interview for DSM-IV and ICD-10. The Journal of clinical psychiatry. 1998;59 Suppl
20:22-33;quiz 4-57.
3. Thompson PM, Gonzalez JM, Singh V, Schoolfield JD, Katz MM, Bowden CL. Principal domains of behavioral psychopathology identified by the Bipolar Inventory of Signs and Symptoms Scale
(BISS). Psychiatry research. 2010;175(3):221-6. doi:10.1016/j.psychres.2009.04.017.


8
